# Do Nonprofit Organizations Experience Precarious Employment? The Impact of NGO Commercialization

**DOI:** 10.1007/s11115-021-00512-w

**Published:** 2021-02-22

**Authors:** Paweł Mikołajczak

**Affiliations:** Department of Money and Banking, Poznań University of Economics and Business, Aleje Niepodległości 10, 61-875 Poznań, Poland

**Keywords:** NGOs, Staff, Precarious employment, Commercialization, Volunteers, E24, E02, J21, L31, O15, D25

## Abstract

The purpose of this article is to examine if Polish non-governmental organizations (NGOs) experience the precarious employment and to identify whether the commercialization of NGOs influences this phenomenon. The study confirms that Polish NGOs experience precarious employment. The greater flexibility a given form of employee engagement provides, the greater the number of NGOs using it. Only a small percentage of organizations employ full-time employees. However, the commercialization does not significantly affect precarious employment in NGOs, moreover it does not impact on the employment of contract employees who had previously worked for the organization as volunteers.

## Introduction

Flexible forms of employing staff are gaining more and more importance and popularity in the market economy, resulting in the redistribution of risk from the state and employers onto citizens. Depending on the specific institutional environment in various sectors of the economy, precarious employment (PE) increases the risk of crowding out protected employees with clear legal status into the sphere of informal employment. There are some differences in the genesis of informal and non-standard forms of employment, but all of them are associated with job insecurity, which allows them to be considered as precarious employment (Volchik and Maslyukova 2019). In the for-profit sector, performance of any type of work on the basis of atypical forms of employment encompasses 3.6% of all employed persons in Poland.

In international literature, the precariat is considered on many levels: political, related to, inter alia, neoliberal principles of shaping the dependence of the state and society (e.g. Byrska 2018); sociological, in relation to the precariat as a new class in society; employment security and its impact on shaping social relationships and the impact of precarious employment on employees, human needs and welfare (e.g. Bessant 2018; Wright 2016); as well as on the economic level. The economic considerations include the macroeconomic approach (e.g. Thelen 2019; Bernards 2017), presenting the consequences of precarious employment for the economy as a whole. In turn, from the point of view of organizations, the research mainly concerns the activities of commercial entities in shaping their competitiveness, dealing with technological changes and cost optimization (e.g. Chernousova 2020; Joynt and Webster 2016).

Unlike the commercial sector, the literature on nonprofit organizations has not yet been thoroughly researched in this field. Much space in international literature has been devoted to the phenomenon of commercialization of nonprofit organizations, the basic assumption and criteria of which is the undertaking of commercial activities by these entities (Mikołajczak 2019). The main purpose of commercializing nonprofit organizations is to strengthen their financial position, increase competitiveness and provide independence from public and private donors in order to fulfill their social mission (e.g. Kerlin and Pollak 2011). In addition to the numerous benefits of commercializing a nonprofit organization, researchers have raised many concerns about the undesirable effects of this approach. Among them, the principal factor is that of distortions related to the social mission, for example mission drift (e.g. Beisland et al. 2019) or mission volatility (e.g. Mikołajczak 2020; D’Espallier et al. 2017), as well as the effects of crowding out private donors or volunteer work (e.g. Hung 2020; Mikołajczak and Bajak 2020).

Since, unlike the commercial sector, the scientific debate about precarious employment in the non-profit sector would appear to be insufficient, it is crucial to study the phenomenon of precarious employment among commercializing NGOs, all the more so because many organizations themselves are struggling with the problems of recruiting people to become involved in their activities, as shown by numerous contemporary studies (e.g. Huntley 2019). The gap in this area became the author’s inspiration to study the phenomenon of PE among NGOs. The purpose of this article is to examine if Polish NGOs experience the precarious employment and to identify whether the commercialization of NGOs influences this phenomenon. A research hypothesis was formulated assuming that the commercialization of Polish NGOs influences precarious employment. To achieve the goal of the article, analysis of the structure of employment forms in the organizations, ANOVA analysis and logistic regression analysis were all carried out.

This article firstly presents the phenomenon of precarious employment. The literature section provides research findings to date with regard to definition and causes of PE; main groups of society suffering from PE as well as different legal forms of precarious employment. The review of the scientific databases indicated a surprisingly very limited number of publications related to the precarious employment in NPOs sector. The literature study in the field made it possible for variables to be selected for the methodological section, which also presents the sample studied and the research procedure. The next - results section presents the structure of precarious employment in NGOs. This part of the article also includes the results of ANOVA analysis and logistic regression analysis, which allowed to compare organizations and for the creation of four models identifying the influence of commercialization of NGOs on the probability of the occurrence of precarious employment. The final section of the article presents conclusions, recommendations for further research and the limitations of the study.

## Literature Review

There is not yet consensus on a definition of precarious employment. A lot of related terms are used interchangeably: the precariat, precarious employment, precarious work, or simply precarity or precariousness (Kreshpaj et al. 2020). The term precariat was originally invented by French sociologists in the 1980s, who used it to define unprotected temporary or seasonal workers as a new social class (Chollet 2006). Beck (2000) identifies precariat as a key aspect of the broader “world risk society” trend characterized by a political economy of insecurity, where Western countries increasingly take on the characteristics associated with the informal economies of poorer countries. In turn Alberti et al. (2018) explains that the spread of the term precariat was the result of the deteriorating situation on the labor market in relation to capital. It is used to describe the expansion of more contingent structures of employment, but also to denote an increase in perceptions of insecurity among workers. Meanwhile, the definition of precarious employment is connected with insecurity, vulnerability, and temporary employment (Grenier et al. 2019; Ervasti and Virtanen 2019). In the taxonomy of precarious employment most studies include social rights, employment conditions and wages (Kalleberg 2009). Some researchers also associate PE with occasional workers or workers with very low incomes, casual workers and fixed-term workers (Bernards 2017). Precarious employment is often characterized by a lack of labour/collective rights and social security (Beck 2000).

Precarious employment is spreading internationally. It has grown in developed and developing countries alike (Günther and Launov 2012). Among the causes of precarious employment at macroeconomic level scholars see significant institutional and technological changes in neoliberal economies (Burrows 2013). For example, Standing (2011) explains reasons of PE in the need to reduce labor costs in order to stop entrepreneurs from relocating production and investments to more profitable places, intended to counteract the slowdown in economic growth. In other findings, the researcher emphasizes that part of this concept is a way of thinking about the labor market, in which the main idea is to increase its flexibility and reduce social security, which is perceived as a burden on employers (Standing 2014). Other scholars argue that informal economy is intrinsically linked to the formal and hence grows in tandem through subcontracting and outsourcing arrangements (Ghose and Chandrasekhar 2015). Some authors indicate that participation in the informal economy may be voluntary because the benefits of informal employment outweigh those of the formal employment (Sapkal and Chhetri 2019). Other studies indicate flexibility of the labor market affects the efficiency of using labor resources, while creating a real risk of a drift towards informal and partial employment (Thelen 2019). In this context, Ferguson (2013), argues that although the appearance of employment contracts safeguarding employees’ interests saw a withdrawal from the regulation of employment relationships on the basis of flexible civil law contracts, the subsequent return to elements of civil law employment regulation can be considered as one of the factors in modern precarious employment.

Scientists focusing on micro level see factors increasing the probability of falling in precarious employment mainly in individual and job-related characteristics. They mainly concern low qualifications, nationality, gender, belonging to socially marginalized groups or young age (Vancea and Utzet 2017). To the social groups that are most at risk of PE researchers include mainly women, young people (with a little professional experience), the less educated and migrants (Volchik and Maslyukova 2019). Women and migrants are the most commonly subject to precarious employment in the US; Asian and European contexts alike (e.g. Chernousova 2020; Roy 2019; Denia and Guillú 2019; Goldin et al. 2006). Findings suggest that entrenched gender expectations around work and family may lead women (regardless of household income) and lower-class men to be most vulnerable to the occurrence of stress (Fan et al. 2019). PE is inevitably characteristic of many migrants’ lives, which are often punctuated by a lack of job security linked to limited material and psychological well-being (Deshingkar 2019). Some studies also point to the problem of the earnings of employees enduring precarious employment, however, the findings in this respect are not clear. For example, Ballafkih (2017) proves that the income of these workers depends mainly on the number of hours they work. This causes fluctuation in their income, which gives them a feeling of insecurity as it is hard to plan their financial situation with a low, varying income. Grimm and Ronneberger (2007) indicate that precarious employment may refer to self-employed and casual employees, or young well-educated people who work for years unpaid, adding, however, that they accept uncertainty as a “normal state of affairs”. Other scholars identified various groups of employees who displayed a high incidence of precariousness, including lower wages (Thomas et al. 2020; Krings 2020). These include full-time employees with low salaries, certain groups of part-time workers, employees with fixed-term contracts and temporary agency workers, as well as self-employed individuals (Julia et al. 2017). Regardless of the individual qualifications of people at risk of PE, these are accompanied by an increase in uncertainty, a lack of a sense of belonging to an organization, increasing anger and frustration, and an increase in behaviors incompatible with generally accepted norms that result from insecurity of employment (e.g. Shammas 2018). It entails a life full of uncertainty where it is difficult to plan, and in which you not only need to change your workplace several times, but also your profession, and even the best position can be lost overnight (Padrosa et al. 2020).

Researchers devote a great deal of space in their work on PE to the elastic legal forms of employment (e.g. Volchnik et al. 2018). These are gaining ever more importance and popularity in the market economy, however they result in the redistribution of risk from the state and employers onto citizens. Gill and Pratt (2008) emphasize that precarious employment applies to all forms of precarious, conditional and flexible work, and includes both illegal, occasional and temporary employment, as well as homeworking, piecework and freelancing. In turn, Standing (2011) indicates an “atypical” and “non-standard” form of work as temporary employment, part-time employment, outsourcing, zero hours contracts, forced unpaid leave, use of interns.

Many scholars emphasize the indisputable crucial role of NGOs in alleviating the challenges that particular groups of societies (mainly form poor countries) face in relation with precarious employment (e.g. Bhutani Vij 2020). However, only several studies indicate examples precarious employment taken place in the non-profit sector. For example, Pentaraki and Dionysopoulou (2019) consider precarious employment among professionals working as social workers in Greek NPOs. The authors argue that this should be understood from the perspective of the international political economy and neoliberal capitalism, which brings an increase in the level of inequality. Similarly, Cunningham et al. (2016) present the results of research against national contexts of recession, neoliberalism and austerity. The authors show that workers in outsourced Scottish and Canadian nonprofit social services are vulnerable to austerity policies and increased precarity. In turn, other researchers see the reasons for the growing employment uncertainty among Canadian NPOs in legislative changes regarding employment and income security programs (Fanelli et al. 2017). In the context of austerity policies, Cunningham et al. (2016) argue that precarious workers are highly vulnerable to expectations that they will ‘volunteer’ at their places of employment, while full-time workers often use unpaid work as a form of resistance. Meanwhile, paid workers in nonprofit organizations very often come from among volunteers who had previously been engaged in the organizations’ activities (Word and Sowa 2017).

### Research Methodology

The article presents a hypothesis which assumes that the commercialization of Polish NGOs influences precarious employment. Data for the analysis was obtained from the Klon/Jawor Association, which partly commissioned Kantar Millward Brown in 2018 to conduct a national survey on a representative sample of 1300 Polish foundations and associations. The research was carried out on a stratified random group of associations and foundations drawn from the REGON GUS register (Main Statistical Office). Data on 1100 NGOs was collected using the CAWI technique (Computer-Assisted Web Interview). The organizations selected for the study received emails inviting them to take part in the study. In addition, they were reminded by phone and encouraged to participate in the study. The respondents were people performing key functions in their organizations and well-versed in their situation, including board members and directors. Data was collected on a confidential basis.

In the study, fout forms of employee involvement in the organization were identified. The first group consists of employees employed under an employment contract. The second form concerns people working permanently and regularly (at least once a week), but not employed under an employment contract, but on repetitive civil contracts, contracts of commission or providing work as part of self-employment for NGOs. Another form of involvement involved regular employees from whom the organization commissioned specific activities from time to time. The fourth form includes people who from time to time have irregularly provided work for the organization (e.g. one-off civil contracts, commission contracts). Table 1 presents the variables adopted for analysis.

**Table 1:** Variables

Variables	Specification
V1	The number of different forms of employment used in the organization
V2	Number of contract employees or permanently and regularly employed under other contracts
V3	Number of people employed permanently and regularly (at least once a week) not under an employment contract, but for example on repetitive civil contracts, contracts of commission or self-employed persons
V4	Number of permanent co-workers from whom some activities were commissioned from time to time (several times a year) for a fee (e.g. in the form of a civil contract, contract of commission or other contract)
V5	Number of people to whom the organization outsourced tasks from time to time, but who do not cooperate with the organization on a regular basis (e.g. only for one-off civil contracts, contracts of commission or other contracts)
V6	Number of people permanently and regularly working under a contract of employment or other forms of employment who previously worked for the organization as volunteers free of charge
V7	NGOs employing staff under a contract of employment
V8	NGOs engaging people permanently and regularly (at least once a week) not on an employment contract, but e.g. on repetitive commission contracts, civil contracts or self-employed persons
V9	NGOs involving permanent associates from whom from time to time (several times a year) the organization commissions activities for a fee (e.g. in the form of a civil contract, contract of commission or other contract)
V10	NGOs involving people whom the organization commissions from time to time for tasks, but who do not cooperate with the organization regularly (e.g. only on one-off civil contracts, commission contracts or other contracts)
E1	NGOs performing commercial activity
E2	NGO revenue

In the first stage of the study, an analysis of the structure of each organization was carried out according to forms of employee involvement, including organizations conducting commercial activity, as well as those not conducting such activity. In the next stage, ANOVA was performed in order to assess the statistical significance of the differences between the two groups of NGOs in terms of the characteristics of precarious employment. Using one-way ANOVA, the differences between NGOs conducting and not conducting business activities were examined across nine variables (V1-V6). First, the compliance of each variable with the normal distribution was tested using the Kolmogorov-Smirnov test (K-S). In cases where the distribution was not normal, the non-parametric Kruskal-Wallis (K-W) test was performed. To verify the assumption of homogeneity of variance, the Brown-Forsythe (B-F) test was used as the groups had different numbers of items. In the absence of homogeneity of variance in both groups, the Welch F test was used to assess the significance of the differences between the groups compared. Analyses were then conducted using logistic regression. The logistic regression model is aimed at defining the probability of NGOs employing staff under the four identified forms of employee involvement used in the organizations. These are presented in Table 1 (V7; V8; V9 and V10), depending on the two selected variables: running commercial activity and NGO revenues. Variable V7 stands for organizations employing staff full-time, while variables V8 to V10 represent organizations implementing precarious employment at different levels, where V8 indicates the high level of precarious employment; V9 medium level and V10 low level of PE. The three levels of precarious employment have been deliberately distinguished because precarious employment is varied in its intensification. Such an approach also makes the results more applicable.

An attempt has been made to quantify and parametrize this likelihood, and as a result, 4 models were obtained. The organization revenue variable was standardized by calculating the natural logarithm of each organization’s revenue. In this way, it was possible to correctly compare individual NGOs. The possibility of predicting NGOs employing staff under each of four identified forms of employee involvement was defined as the probability of the NGOs falling, on the basis of the survey results, into one of two binary classes (0 – use of the given form did not take place, 1 – NGOs employ staff under the given form). The suitability of the models obtained from the data was evaluated by performing a χ2 test. It was assumed that there would be the risk of a 5% error of inference as well as an associated significance level of *p* < 0.05, indicating the existence of statistically significant dependencies. The quality of the logistic regression models constructed was assessed using the Hosmer-Lemeshow (H-L) test, the zero hypothesis of which is a good fit for the models. This test compares the values of the calculated probability with the observed values of the investigated phenomenon of NGOs employing staff under each of four identified forms of employee involvement. While verifying the correctness of the models, a collinearity analysis of explanatory variables was also performed, the effect of which is expressed by the VIF factor (variance inflation factor). Assessment of factors affecting the likelihood of NGOs employing staff under a specific form of employee involvement was also conducted based on the unit odds ratio (OR_*i*_), which takes on higher, lower or zero values.

## Results

Only 23.7% of NGOs employed under a contract of employment. 30.5% of NGOs used employees on a permanent and regular basis not employed under a contract of employment, but on repetitive civil contracts, commission contracts or through providing work to the NGO as part of their own business. Over a third of the NGOs (36.8%) had permanent employees whom they occasionally assigned specific activities to. In turn, almost half of the NGOs (47.8%) from time to time outsourced work to people with whom they did not cooperate regularly (see Table 2).

**Table 2:** Analysis of the popularity of employment forms used by NGOs

Variables	Total (in %)
V7	23.7
V8	30.5
V9	36.8
V10	47.8

The use of more or less flexible forms of cooperation under a civil contract or a commission contract, or the outsourcing of tasks to persons conducting business activity took place much more often in the case of NGOs that employed staff under a contract of employment. Among the organizations employing on the basis of an employment contract, 63.2% engaged employees under repetitive civil contracts, commission contracts or outsourced tasks to persons who regularly undertook specific activities for NGOs as part of their own business operations. Also, these NGOs much more often engaged permanent associates from whom they occasionally commissioned activities for payment than organizations without employees under a contract of employment. Similarly, a high percentage of NGOs that employed under a contract of employment from time to time commissioned activities for a fee from persons who did not cooperate with the organization regularly (see Table 3).

**Table 3:** Analysis of the popularity of flexible forms of employment among NGOs employing and not employing employees under a contract of employment

Variables	NGOs employing under a contract of employment (in %)	NGOs not employing under a contract of employment (in %)
V8	63.24	20.31
V9	65.88	27.63
V10	69.41	41.08

The conducting of commercial activity by NGOs promotes greater popularity of all forms of employment. Analysis of the popularity of flexible forms of employment among NGOs performing commercial activity and NGOs not conducting such activity shows that among units from both groups that employ employees under a contract of employment, the popularity of more flexible forms of employment is at a very similar level. There are clear differences in the popularity of flexible forms of employment between NGOs performing and not performing commercial activity that do not employ on the basis of an employment contract (see Table 4).

**Table 4:** Analysis of the popularity of flexible forms of employment among NGOs conducting and not conducting commercial activity

Variable	NGOs conducting commercial activity			NGOs not conducting commercial activity		
	NGOs employing under a contract of employment (in %)	NGOs not employing under a contract of employment (in %)	Total (in %)	NGOs employing under a contract of employment (in %)	NGOs not employing under a contract of employment (in %)	Total (in %)
V8	66.67	32.04	48.50	61.89	19.09	27.57
V9	69.79	40.78	55.00	64.34	26.26	33.87
V10	73.96	52.43	63.00	67.62	39.90	45.35

Over a third of the organizations do not employ any employees (35.1%). Among these organizations, a much larger percentage are entities not conducting commercial activity (38.2%), while entities performing such activity constitute only 16%. As the number of forms of employment increases, the percentage of organizations that confirm their use decreases. Such regularity cannot be seen when the results for the segment of organizations conducting commercial activity are analyzed (see Table 5).

**Table 5:** Analysis of the number of forms of employment used by NGOs conducting and not conducting commercial activity

Number of forms of employment	NGOs conducting commercial activity (in %)	NGOs not conducting commercial activity (in %)	Total (in %)
0	16.00	38.24	35.14
1	21.50	25.22	24.70
2	17.50	16.01	16.21
3	22.00	12.85	14.13
4	23.00	7.68	9.81
Total	100	100	100

Using one-way ANOVA analysis, the differences between NGOs conducting and not conducting commercial activity were examined in terms of the number of different forms of employment used (V1). Firstly, a test was carried out on the compliance of the variable distribution with the normal distribution, in which a Kołmogorov-Smirnov (K-S) d test value of 0.212 was obtained (*p* < 0.01). Therefore, a basis was obtained for rejecting the hypothesis on the normal distribution of the examined feature (see Table 6).

**Table 6:** ANOVA analysis

Assessment category	H K-W test value	*p*	F B-F test value	*p*	F Test	*p*	F Welch test value	*p*
V1 – E1	67.8152	0.0000	8.8750	0.0029	–	–	68.6482	0.0000
V2 – E1	2.2881	0.1304	1.4129	0.2354	1.6049	0.2061	–	–
V3 – E1	0.0908	0.7631	2.8601	0.0915	2.6347	0.1053	–	–
V4 – E1	9.5078	0.0020	3.4384	0.0643	–	–	4.3262	0.0396
V5 – E1	12.7137	0.0004	0.0514	0.8206	0.0993	0.7527	–	–
V6 – E1	0.0228	0.8799	3.6969	0.0551	3.0338	0.0821	–	–

Subsequently, a non-parametric Kruskal-Wallis (K-W) test was carried out, whose H statistic was 67.8, with a computer significance level of *p* < 0.05. Therefore, there are grounds to reject the assumption that there are no significant differences between the average scores for NGOs conducting commercial activity and those not performing such activity. The B-F test was used to check the assumption of homogeneity of variance due to the unequal number of groups. The number of NGOs performing commercial activity differed from the number of organizations that did not perform such activity. The test result indicated a lack of homogeneity of variance in both groups (F statistic = 8.87, at *p* < 0.05). The Welch F test was used to assess the means. The value of the empirical statistics of the Welch F test was 68.64, at *p* < 0.05. Therefore, a basis was obtained to reject the assumption that there were no significant differences between the average values of the examined feature - the number of forms of employment used. There are statistically significant differences between NGOs conducting and not performing commercial activity at the level of α = 5%. NGOs performing commercial activity use more forms of employment compared to those not conducting such activity (see Table 6).

The test results allow similar conclusions to be formulated regarding the differences between NGOs conducting economic activity and those not conducting such activity in terms of the number of permanent co-workers who from time to time were commissioned for specific activities for a fee (V4). The number of contract employees or permanently and regularly working under other contracts (V2) is not a feature that differentiates between NGOs conducting and not conducting commercial activity. In a test of the compliance of the variable distribution with the normal distribution, a K-S d test value of 0.40 (*p* < 0.01) was obtained, which gives a basis for rejecting the hypothesis on the normal distribution of the examined feature (see Table 6). Subsequently, a non-parametric K-W test was carried out, whose H statistic was 2.2886, at *p* > 0.05. Therefore, there are no grounds for rejecting the assumption that there are no significant differences between the average scores for NGOs conducting commercial activity and those not conducting such activity. The B-F test was used to check the assumption of homogeneity of variance due to the unequal number of groups. The number of NGOs performing commercial activity differed from the number of organizations that did not perform such activity. The result of the test indicated homogeneity of variance in both groups (F statistic = 1.41, at p > 0.05), hence the F test was used to assess the means, giving a value of 1.60, at *p* > 0.05.

Therefore, no bases were found for rejecting the assumption that there were no significant differences between the average values ​​of the examined feature - the number contract employees or permanently and regularly employed under other contracts. With regard to the number of contract employees and permanent and regular employees employed under other forms of employment, there are no statistically significant differences between NGOs conducting and not conducting commercial activity (see Table 6).

The test results allow similar conclusions to be formed about the lack of differences between NGOs conducting economic activity and those not conducting such activity in terms of the following factors; the number of people employed permanently and regularly not on an employment contract, but, for example, on repetitive commission contracts, civil contracts or self-employed persons (V3); the number of persons to whom the organization outsourced tasks from time to time, but who did not cooperate with the organization on a regular basis (V5); the number of people working permanently and regularly under a contract of employment or other forms of employment who had previously worked for the organization as volunteers (V6) (see Tables 6).

As mentioned above, logistic regression was carried out in order to establish which factors are significant and influence an NGO in employing staff under the indicated four forms of employee involvement (V7, V8, V9, V10). The first model of the logistic regression analysis used the binary variable V7, that is NGOs employing contract employees, together with the explanatory variables E1 and E2. Both of these variables were shown to determine the probability of an NGO employing staff under an employment contract. For other models indicating the probability of NGOs making use of precarious employment (V8-V10), the explanatory variable E1 is not statistically significant. The models indicate, however, that variable E2 does determine the probability of an NGO employing under the identified forms of employee involvement (V8; V9 and V10). The odds ratio (ORi) for all continuous predictors is greater than 1, which means that the variables have a positive impact on the four forms of employment under study (see Table 7).

**Table 7:** Parameters of independent variables of the logistic regression models

Variable	Model 1	Model 2	Model 3	Model 4
	Coefficient			
E1	0.479*	–	–	**–**
E2	0.9618 **	0.6843 **	0.5222**	0.3671**

*p < 0.05

**p < 0.01

Model 1

The logistic regression analysis demonstrated that E1 and E2 caused an increase in the probability of NGOs employing staff under an employment contract, as its directional coefficient was 0.479; 0.9618. Model 1 achieved a χ2 test value of 249.29, with a *p* value of 0.00, which means that it is statistically significant. The model also accurately reflects actual data (the H-L test indicated a *p* value of 0.138). The model demonstrates that the likelihood of an NGO employing staff under an employment contract increases with the NGOs commercialization and revenue. The model is therefore described by the following formula 1:

**Formula 1.** Equation of logistic regression for V7

$$ V7=\frac{\mathit{\exp}\left(-\mathrm{12,240}+\mathrm{0,479}\ \mathrm{E}1+0,9618\ \mathrm{E}2\right)}{1+\mathit{\exp}\left(-\mathrm{12,240}+\mathrm{0,479}\ \mathrm{E}1+0,9618\ \mathrm{E}2\right)}\kern0.5em , $$

Model 2

The logistic regression analysis demonstrated that E2 caused an increase in the probability of NGOs engaging people permanently and regularly (at least once a week), not on an employment contract but e.g. on repetitive commission contracts, civil contracts or self-employed persons (V8), as its directional coefficient was 0.6843. Model 2 achieved a χ2 test value of 235.01, with a *p* value of 0.00, which means that it is statistically significant. The model also accurately reflects actual data (the H-L test indicated a *p* value of 0.074). It indicates that the likelihood of an NGO engaging people permanently and regularly, not on an employment contract but e.g. on repetitive commission contracts, civil contracts or self-employed persons increases with revenue (see formula 2).

**Formula 2.** Equation of logistic regression for V8

$$ V8=\frac{\mathit{\exp}\left(-\mathrm{8,302}+0,6843\ \mathrm{E}2\right)}{1+\mathit{\exp}\left(-\mathrm{8,302}+0,6843\ \mathrm{E}2\right)}\kern0.5em , $$

Model 3

The logistic regression analysis demonstrated that E2 caused an increase in the probability of NGOs involving permanent associates from whom from time to time the organization commissions activities for a fee (V9), as its directional coefficient was 0.5222. Model 2 achieved a χ2 test value of 198.32, with a p value of 0.00, which means that it is statistically significant. The model also accurately reflects actual data (the H-L test indicated a p value of 0.063). It demonstrates that the likelihood of an NGO involving permanent associates from whom from time to time the organization commissions activities for a fee increases with revenue (see formula 3).

**Formula 3.** Equation of logistic regression for V9

$$ V9=\frac{\mathit{\exp}\left(-\mathrm{6,101}+0,5222\ \mathrm{E}2\right)}{1+\mathit{\exp}\left(-\mathrm{6,101}+0,5222\ \mathrm{E}2\right)}\kern0.5em , $$

Model 4

The logistic regression analysis demonstrated that E2 caused an increase in the probability of NGOs involving people whom the organization commissions from time to time for tasks, but who do not cooperate with the organization regularly (V10), as its directional coefficient was 0.3671. Model 2 achieved a χ2 test value of 137.04, with a p value of 0.00, which means that it is statistically significant. The model also accurately reflects actual data (the H-L test indicated a p value of 0.056). The model demonstrates that the likelihood of an NGO engaging people permanently and regularly not on an employment contract, but e.g. on repetitive commission contracts, civil contracts or self-employed persons increases with revenue (see formula 4).

**Formula 4.** Equation of logistic regression for V10

$$ V10=\frac{\mathit{\exp}\left(-\mathrm{3,856}+0,3671\ \mathrm{E}2\right)}{1+\mathit{\exp}\left(-\mathrm{3,856}+0,3671\ \mathrm{E}2\right)}\kern0.5em , $$

## Conclusions

The extant literature on precarious employment among NGOs is very sparse. Also, there has been very limited theoretical and empirical exploration of how commercialization affects PE among NGOs. This study makes three key contributions. First, through an empirical analysis, it introduces the issue of precarious employment in NGOs into the scientific discussion. The results of the study confirm that Polish NGOs experience the precarious employment. Only slightly more than a fifth of the NGOs surveyed employed contract employees. The vast majority used flexible civil-law contracts, and of these almost half exclusively engaged people occasionally on one-off commission contracts and on civil contracts. The study showed that the greater the flexibility of a given form of employee involvement, the higher the percentage of NGOs using it. Secondly, empirical verification of the hypothesis put forward in the article has also made it possible to develop scientific knowledge by proving that the commercialization of NGOs does not significantly affect precarious employment. The commercialization factor only has a significant impact among organizations with full-time employees. Moreover, the size of the budget of organization, measured by the revenues it earns, has a significant positive effect on all forms of employment. The results of this study also confirm that the commercialization of NGOs does not impact on the employment of people who work permanently and regularly under a contract of employment or other forms of employment, and who had previously worked for the organization free of charge as volunteers. Thirdly, the article contributes to knowledge and the theory of precarious employment which so far has been considered primarily in relation to commercial entities, among which economic efficiency is the basis of their market activity. The article identifies PE in NGOs belonging to nonprofits sector. Although they very often counteract the negative consequences of precarious employment in commercial sector, they also struggle with insecure employment themselves.

Finally, the study results are significant globally, not only for other V4 and Central and Eastern European countries but also for many developing countries worldwide where the problem of precarious employment seems particularly relevant today in the face of the COVID-19 pandemic. The relatively poor condition of NGO linked to post-communist experiences that strongly shaped the nonprofit sector, civil society, and the possibilities for NGOs to function and the economic slowdown caused by COVID-19, may, as in commercial entities, force organizations to implement cutbacks, also in the area of employment.

This article opens up a wide field for continuing research, primarily on precarious employment determinants in the non-profit sector. In view of the negative consequences of this phenomenon, it is also necessary to search for answers to the questions which activities and instruments should be used to maintain employment in organizations and the level of work required to ensure their efficient functioning. This applies to both paid employees and volunteers.

Considering limitations of this study, one should bear in mind that the legal forms of employment may differ in different countries. The impact of commercialization may vary depending on the legal, institutional and social conditions of the organization in individual economies.
